# Plasma Membrane Phosphatidylinositol 4,5 Bisphosphate Is Required for Internalization of Foot-and-Mouth Disease Virus and Vesicular Stomatitis Virus

**DOI:** 10.1371/journal.pone.0045172

**Published:** 2012-09-28

**Authors:** Ángela Vázquez-Calvo, Francisco Sobrino, Miguel A. Martín-Acebes

**Affiliations:** 1 Centro de Biología Molecular “Severo Ochoa” (UAM/CSIC), Cantoblanco, Madrid Spain; 2 Centro de Investigación en Sanidad Animal, INIA, Valdeolmos, Madrid, Spain; The Scripps Research Institute, United States of America

## Abstract

Phosphatidylinositol-4,5-bisphosphate, PI(4,5)P_2_, is a phospholipid which plays important roles in clathrin-mediated endocytosis. To investigate the possible role of this lipid on viral entry, two viruses important for animal health were selected: the enveloped vesicular stomatitis virus (VSV) − which uses a well characterized clathrin mediated endocytic route − and two different variants of the non-enveloped foot-and-mouth disease virus (FMDV) with distinct receptor specificities. The expression of a dominant negative dynamin, a PI(4,5)P_2_ effector protein, inhibited the internalization and infection of VSV and both FMDV isolates. Depletion of PI(4,5)P_2_ from plasma membrane using ionomycin or an inducible system, and inhibition of its *de novo* synthesis with 1-butanol revealed that VSV as well as FMDV C-S8c1, which uses integrins as receptor, displayed a high dependence on PI(4,5)P_2_ for internalization. Expression of a kinase dead mutant (KD) of phosphatidylinositol-4-phosphate-5-kinase Iα (PIP5K-Iα), an enzyme responsible for PI(4,5)P_2_ synthesis that regulates clathrin-dependent endocytosis, also impaired entry and infection of VSV and FMDV C-S8c1. Interestingly FMDV MARLS variant that uses receptors other than integrins for cell entry was less sensitive to PI(4,5)P_2_ depletion, and was not inhibited by the expression of the KD PIP5K-Iα mutant suggesting the involvement of endocytic routes other than the clathrin-mediated on its entry. These results highlight the role of PI(4,5)P_2_ and PIP5K-Iα on clathrin-mediated viral entry.

## Introduction

Phosphatidylinositols (PIs) and their phosphorylated derivatives are low abundant lipids in cellular membranes (<10% of total phospholipids) that have been revealed as key membrane components, particularly for membrane traffic [1]. One of these lipids, phosphatidyilinositol-4,5-bisphosphate (PI(4,5)P_2_), which is mostly localized in the internal hemimembrane of the plasma membrane, participates in regulation of a variety of cellular processes such as generation of membrane curvature, fission of endosomes, exocytosis, and binding to different effectors of clathrin-dependent endocytosis as well as at actin regulator proteins [1], [2], [3], [4]. In this way, depletion of PI(4,5)P_2_ from plasma membrane has been shown to inhibit clathrin-mediated endocytosis [2], [3], [4], [5], [6], [7]. In this endocytic route, clathrin-coated pits (CCPs) are assembled at the plasma membrane from cytosolic coat proteins. Upon capture of transmembrane receptor molecules CCPs invaginate to maturate into clathrin-coated vesicles (CCVs) [8]. Recent reports have shown that synthesis of PI(4,5)P_2_ is the major determinant of PI(4,5)P_2_ availability for CCP initiation and nucleation by contributing to progression beyond the endocytosis checkpoint and stabilization of nascent CCPs [2], [3], [4], [5], [6], [9], [10]. Although late states of CCP maturation to CCV do not require the synthesis of PI(4,5)P_2_, the presence of this lipid is necessary to bind proteins involved in CCV scission [2], 11. As the other PIs, PI(4,5)P_2_ carries out these regulatory functions by binding to different effector proteins through well characterized domains [1]. It is suggested that PI(4,5)P_2_ levels regulate CCP assembly, whereas localized turnovers of this phospholipid can control multiple stages in CCV formation [2], [12]. The major route for PI(4,5)P_2_ synthesis is the phosphorylation of PI4P by type I phosphatidylinositol-4-phosphate-5-kinase (PIP5K-I) [13]. Among the three isoforms reported for this enzyme (α, β and γ) [13], PIP5K-Iα is the major isoform involved in the regulation of clathrin-dependent endocytosis [2].

Proteins interacting with PIs (e.g Rab proteins, dynamin) have been involved in the entry of multiple viruses [14], [15], [16], [17], [18], [19], [20], thus pointing the importance of specific PIs in several steps for viral progression. However, a direct involvement of PIs, and specifically of PI(4,5)P_2_, in viral entry has been poorly evaluated, and the evidence for this is limited to the Human immunodeficiency virus type-1 (HIV-1) entry [21]. Indeed, HIV-1 binding to the plasma membrane through Env-gp120 actives an specific isoform of PIP5K protein, increasing the production of PI(4,5)P_2_. In addition, PI(4,5)P_2_ is required for late steps of HIV-1 and HIV-2 infection to promote the localization of Gag protein on plasma membrane during viral assembly [22], [23].

In the present study, we addressed the role of PI(4,5)P_2_ on the internalization of non-enveloped as well as of enveloped viruses. For this purpose, two important pathogens for animal health, causing clinically indistinguishable diseases, were selected: foot-and-mouth disease virus (FMDV) and vesicular stomatitis virus (VSV). FMDV is a small, non-enveloped virus responsible for a highly contagious disease affecting cloven-hoofed animals [24]. FMDV initiates infection of cultured cells via different α_v_ integrins [25], [26], [27], [28], although receptors different from integrins can be used by FMDV variants selected upon passages in cultured cells [29], [30]. FMDV isolates that recognize integrins as cellular receptor utilize CCPs to enter cultured cells [31], [32], [33], while FMDV variants using heparan sulphate (HS) proteoglycans as receptor instead of integrins are internalized via caveolae [34]. To compare the PI(4,5)P_2_ requirements derived from the use of distinct cellular receptors, two different FMDV isolates were included in the study: C-S8c1 that is dependent on integrins for infection [35] and MARLS, a C-S8c1 derivative that has acquired the ability to enter cultured cells using HS and other not well characterized receptor(s) [29], [36]. On the other hand, VSV is an enveloped virus that has become a widely used system for the study of the clathrin-mediated endocytosis of viruses [37], [38], [39], [40].

Our results showed that dynamin − a PI(4,5)P_2_ effector − was a common requirement for infection of VSV and the two FMDVs analyzed. In contrast, while endocytosis of both FMDV C-S8c1 and VSV was highly dependent on plasma membrane PI(4,5)P_2_, that of FMDV MARLS showed a lower dependence on this phospholipid.

## Results and Discussion

### Functional Requirement of Dynamin for FMDV and VSV Infection

**Figure 1:**
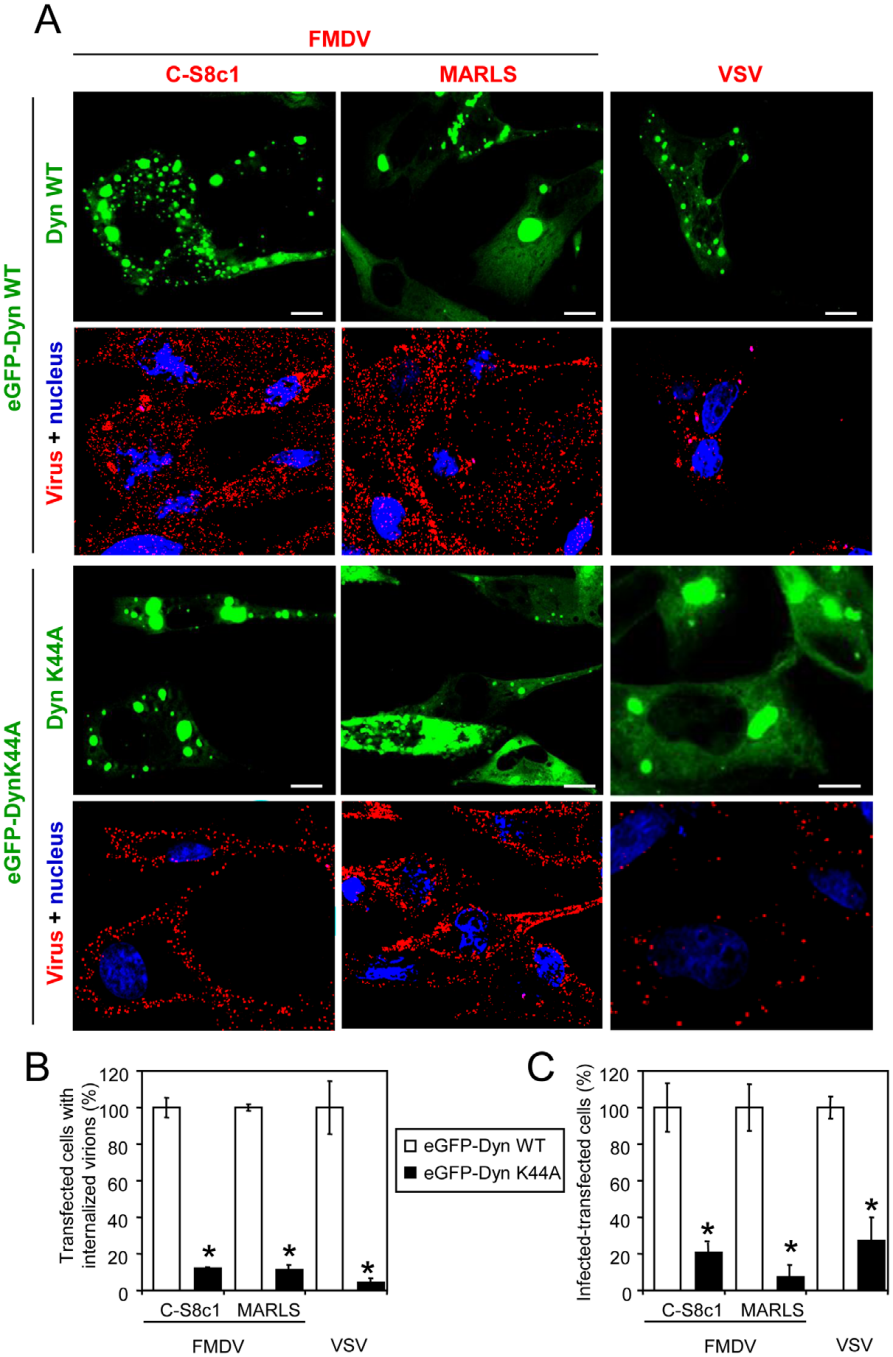
Functional requirement for dynamin of FMDV and VSV infection. (A) BHK-21 cells transfected with eGFP fused to WT or a DN version of dynamin (eGFP-Dyn WT and eGFP-Dyn K44A, respectively) and 24 h later were incubated with the different FMDV variants (C-S8c1 and MARLS) or VSV (MOI of 70 PFU/cell) for 25 min and processed for immunofluorescence. Nuclei were stained using ToPro-3 (blue). GFP and viruses are shown in green and red, respectively. Bar: 10 µm. (B) BHK-21 cells transfected and infected as in (A). The graph represents the percentage of cells that showed internalized virus, determined as described in Materials and Methods. At least 100 transfected cells per coverslip were scored in each assay (3 coverslip). (C) BHK-21 cells were electroporated with a plasmid encoding eGFP-Dyn WT as control, or eGFP-Dyn K44A. At 24 h post-electroporation, monolayers were infected with the corresponding virus (MOI of 1 PFU/cell). Cells were fixed and processed for immunofluorescence at 7 h post-infection. Bars represent the mean percentage of transfected and infected cells ± SD, normalized to the level of infection of cells expressing the eGFP-Dyn WT. Statistically significant differences between cells transfected with eGFP-Dyn WT or K44A are indicated by an asterisk (ANOVA P≤0.05).

PI(4,5)P_2_ is involved in several cellular events as a result of its binding to different partners [1], [4], [41], [42], [43], [44], [45]. One of these proteins is the GTPase dynamin that works in endocytosis as a regulatory molecule and as a component of the fission machinery [46]. This prompted us to analyze the effect of the expression of a dominant negative (DN) form of dynamin (Dyn K44A) [47] on the entry of two FMDV isolates with different receptor specificities (C-S8c1 and MARLS). In this study, VSV was included as a positive control since it is well documented that dynamin is necessary for its entry [37], [38], [40]. BHK-21 cells were transfected with the corresponding plasmid, incubated with the viruses, and analysed by confocal microscopy as described [32], [48]. In cells expressing the WT dynamin, the viral particles were located inside the cells. However, the virions were observed at the cellular periphery in cells expressing the DN dynamin (Fig. 1A). When the percentage of cells with internalized viral particles was estimated by confocal microscopy,similar values were observed in control cells infected with VSV, C-S8c1 and MARLS, while the percentages were significantly reduced in cells expressing DN dynamin (12%, 11% and 4%, respectively), indicating that all viruses tested required dynamin for cell entry (Fig. 1B). In addition, expression of DN dynamin significantly reduced the percentage of transfected-infected cells for C-S8c1, MARLS and VSV (Fig. 1C), and no significant differences between the degrees of inhibition of the three viruses were noticed. Overall, these results indicate the functional requirement of this PI(4,5)P_2_-interacting protein for the entry and infection of the three viruses tested.

### Effect of Depletion of PI(4,5)P_2_ on FMDV and VSV Internalization

**Figure 2:**
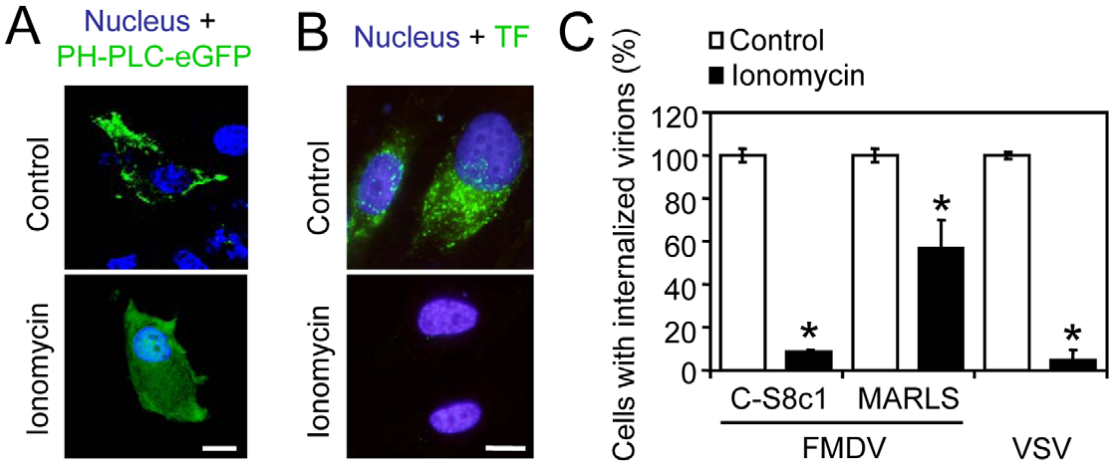
Effect of PI(4,5)P_2_ depletion by ionomycin on FMDV and VSV internalization. (A) Visualization of PI(4,5)P_2_ depletion from plasma membrane. BHK-21 cells transfected (24 h) with PH-PLC-eGFP, encoding a reporter protein for PI(4,5)P_2_ fused to GFP (green), were treated or not with 5 µM ionomycin 30 min and then fixed and observed by confocal microscopy. Nuclei were stained using ToPro-3 (blue). Bar 10 µm. (B) Treatment with ionomycin inhibits clathrin-mediated endocytosis. BHK-21 cells, treated with ionomycin as in (A), were incubated with Alexa Fluor 488-labelled TF (green) for 5 min in the presence of the drug and extracellular TF was eliminated by acid wash as described [32]. Cells were fixed and nuclei were stained using DAPI (blue). Bar: 10 µm. (C) Inhibition of the ability of cells to internalize FMDV and VSV upon ionomycin treatment. Cells treated with ionomycin as in (A) were incubated with the different FMDV variants (C-S8c1 and MARLS) or with VSV (MOI of 70 PFU/cell) for 25 min in the presence of ionomycin. Cells were fixed and processed for immunofluorescence to stain viral particles as described in Materials and Methods. Bars represent the mean percentage of cells with internalized virions ± SD, normalized to the level of cells with internalized virions in control samples. At least 500 cells per coverslip were scored for each case (3 coverslips). Asterisks denote statistically significant differences (ANOVA P≤0.05).

It has been documented that ionomycin treatment reduces the levels of PI(4,5)P_2_ by activation of phospholipase C [4]. BHK-21 cells were transfected with a reporter plasmid (PH-PLC-eGFP) [49] that expresses a fusion protein that binds and allows detection of this phospholipid by fluorescence microscopy [44]. Under control conditions, fluorescence was concentrated at the plasma membrane. However, in cells treated with 5 µM ionomycin as described [4], a cytoplasmic distribution of the reporter for PI(4,5)P_2_ was observed (Fig. 2A). This result confirmed that ionomycin treatment induced a depletion of PI(4,5)P_2_ from plasma membrane, which resulted in a cytoplasmic relocation of the fluorescent reporter protein. The effect of this drug on clathrin-mediated endocytosis was further analyzed by using fluorescent transferrin (TF), a marker of clathrin-mediated endocytosis [50]. In contrast to non treated cells, those treated with ionomycin displayed a reduction in TF internalization (Fig. 2B). As these results supported the conclusion that ionomycin treatment affected clathrin-mediated endocytosis, the effect of this drug on virus internalization was tested. Thus, BHK-21 cells were treated with ionomycin, incubated with the viruses and the proportion of cells that internalized viral particles was determined by immunofluorescence and confocal microscopy. As expected, ionomycin treatment inhibited (reduction by 90%) the internalization of VSV particles (Fig. 2C), whose entry through clathrin-mediated endocytosis is well characterized [37], [38], [39], [40], and a similar reduction was observed for FMDV C-S8c1 that enters into the cells via CCPs [32]. However, the inhibition was lower for FMDV MARLS (only a reduction about 40%), which has the ability to use cellular receptors other than integrins. These results highlight that depletion of PI(4,5)P_2_ from plasma membrane inhibits endocytosis of viruses that use CCV, and reveal that the different internalization pathway followed by C-S8c1 and MARLS FMDV variants can modulate PI(4,5)P2 requirement. Ionomycin treatment induced a significant reduction (close to 30%) on cell viability (Fig. S1A). To rule out that the inhibitory effect of this drug on viral entry could be related to major metabolic alterations, the effect of modulation of PI(4,5)P_2_ was further assayed using drugs with lower cytotoxicity.

### Effect of Inhibition of PI(4,5)P_2_ Synthesis on FMDV and VSV Internalization

**Figure 3:**
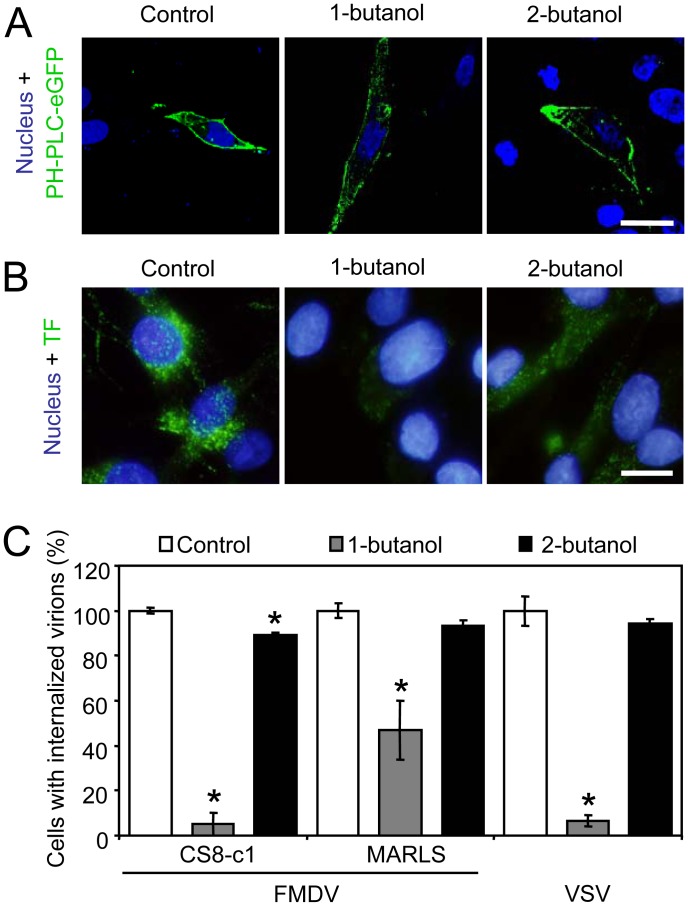
Effect of blocking *de novo* synthesis of PI(4,5)P_2_ with 1-butanol on FMDV and VSV internalization. (A) BHK-21 cells transfected (24 h) with PH-PLC-eGFP (green) were treated or not with 1.5% 1-butanol or 2-butanol for 5 min and then fixed and observed by confocal microscopy. Nuclei were stained using ToPro-3 (blue). Bar 10 µm. (B) Treatment with 1-butanol inhibits clathrin-dependent endocytosis. BHK-21 cells were treated as in (A) were incubated with fluorescent TF and processed as described in the legend of Fig. 1. Bar: 10 µm. (C) Reduction of the ability of cells to internalize FMDV and VSV upon 1-butanol treatment. Cells treated as in (A) were incubated with the different FMDV variants (C-S8c1 and MARLS) or VSV (MOI of 70 PFU/cell) for 25 min in the presence of the drugs. Bars represent the mean percentage of cells with internalized virions ± SD, normalized to the level of cells with internalized virions in control samples. At least 500 cells per coverslip were scored for each case (3 coverslips). Asterisks denote statistically significant differences (ANOVA P≤0.05).

Rather than causing PI(4,5)P_2_ depletion from plasma membrane, as described for ionomycin, primary alcohols, like 1-butanol, promote phospholipase D (PLD) to generate phosphatidylalcohols instead of phosphatidic acid (PA), leading to a lower activation of PIP5Ks and consequently inhibiting PI(4,5)P_2_ synthesis and CCP assembly [5]. On the other hand, this effect does not occur in the presence of secondary alcohols such as 2-butanol [5]. Treatment with either 1.5% 1-butanol or 2-butanol did not affect the distribution pattern observed for the PI(4,5)P_2_ reporter (Fig. 3A), confirming that none of these alcohols produced a significant depletion of PI(4,5)P2 from plasma membrane. However, treatment with 1-butanol reduced the ability of BHK-21 cells to internalize TF, indicating a requirement for *de novo* synthesis of PI(4,5)P_2_ of clathrin-mediated endocytosis (Fig. 3B). As expected, treatment with 2-butanol did not result in reduction of TF internalization, confirming the specificity of the inhibition of clathrin-mediated endocytosis by 1-butanol. In contrast to ionomycin, treatment with either 1 or 2-butanol did not significantly affect cell viability (Fig. S1B), confirming that the reduction of clathrin-mediated endocytosis by 1-butanol was not related to major toxic effects of the drug. Regarding viral entry, treatment with 1-butanol reduced by 90% the internalization of C-S8c1 and VSV. Conversely, it only reduced MARLS internalization by 50%, suggesting that the different receptor used by MARLS and C-S8c1 for cell entry can modulate the requirement of PI(4,5)P2 synthesis. The specificity of this inhibition was confirmed as treatment with 2-butanol resulted in a limited reduction by 10% in the internalization of each of the three viruses analyzed (Fig. 3C). These results indicate that FMDV and VSV require synthesis of PI(4,5)P_2_ at the plasma membrane for internalization, and again suggest differences between MARLS and C-S8c1 internalization pathways.

### Effect of Induced Depletion of PI(4,5)P_2_ from the Plasma Membrane on FMDV and VSV Internalization

**Figure 4:**
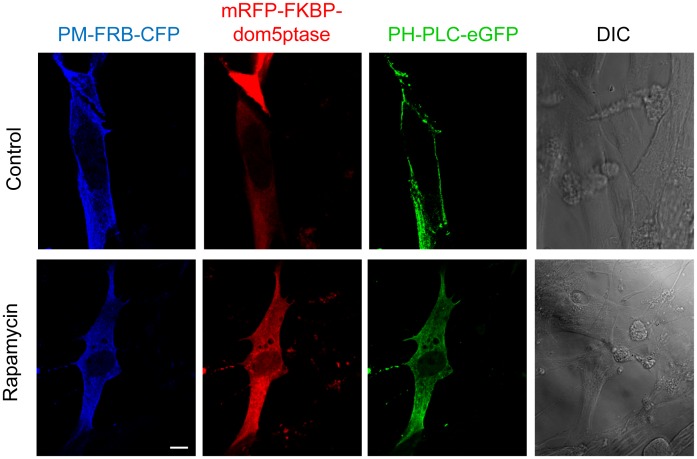
Depletion of PI(4,5)P_2_ from plasma membrane after rapamycin-induced membrane targeting of an inositol 5-phosphatase. BHK-21 cells were cotransfected with PM-FRB-CFP (blue), mRFP-FKBP-dom5ptase (red) and PH-PLC-eGFP (green) plasmids using Lipofectamine Plus. At 24 h post-transfection, cells were treated with 10 nM rapamycin (10 min) to induce the depletion of PI(4,5)P_2_ from plasma membrane. Then cells were fixed and observed by confocal microscopy. A representative example of a co-transfected cell is shown. See text for details regarding the inducible system for PI(4,5)P_2_ depletion. Differential interference contrast (DIC) images are also shown. Bar: 10 µm.

To confirm the results obtained with pharmacological inhibitors, the effect of PI(4,5)P_2_- targeted depletion with an inducible system [45] was analyzed. The approach used, which has been proven to be highly specific [4], [51], [52], is based on the conditional recruitment to plasma membrane of an inositol 5-phosphatase – fused to red fluorescent protein (RFP)– by its rapamycin-induced heterodimerization with a membrane-targeted, rapamycin-binding domain of mTOR fused to the cyan fluorescent protein (CFP) [45]. In this way, only when rapamycin is added to the culture medium, both fusion proteins interact and the phosphatase is recruited to plasma membrane causing a targeted depletion of PI(4,5)P_2_. First, we evaluated that this system induced the depletion of PI(4,5)P_2_ from the plasma membrane when rapamycin was added to BHK-21 cells (Fig. 4). To this end, cells were cotransfected for 24 h with plasmids encoding phosphatase (mRFP-FKBP-dom5ptase), membrane anchored rapamycin-binding domain (PM-FRB-CFP) and PH-PLC-eGFP (to detect PI(4,5)P_2_). Then, cells were treated with rapamycin for 10 min to induce the depletion of PI(4,5)P_2_ from plasma membrane. As expected, when rapamycin was added the fluorescence of PI(4,5)P_2_ reporter protein was relocated from plasma membrane to the cytoplasm (Fig. 4). Next, cells cotransfected with plasmids mRFP-FKBP-dom5ptase and PM-FRB-CFP, were treated with rapamycin to induce the PI(4,5)P_2_ depletion, and then incubated with the viruses (Fig. S2). About 89% of the cells expressing both plasmids in the absence of rapamycin were able to internalize C-S8c1 (Table 1). However, when rapamycin was added, only 12% of cotransfected cells were shown to internalize C-S8c1 particles. Addition of rapamycin similarly reduced the number of cells internalizing VSV (from 88% in control cells to 15% in rapamycin-treated cells for VSV). On the other hand, targeted depletion of PI(4,5)P_2_ only slightly reduced the percentage of cells internalizing MARLS (from 83% in control cells to 76% in rapamycin treated cells) (Table 1). Rapamycin alone had no effect on viral internalization of any of the three virus tested, since treatment with rapamycin of untransfected cells or cells only transfected with one plasmid did not reduce the percentages of cells that internalized the viral particles (data not shown). Overall, these results support those previously obtained with pharmacological treatments, indicating that internalization of FMDV C-S8c1 and VSV strongly depends on plasma membrane PI(4,5)P_2_ phospholipids, while MARLS internalization is less sensitive to PI(4,5)P_2_ depletion. These differences could be explained by the usage of an alternative dynamin-dependent endocytic pathway, such as caveolae, for MARLS internalization, since caveolae-mediated endocytosis can rely on dynamin function [53] and is less sensitive to PI(4,5)P_2_ depletion than clathrin-mediated endocytosis [54]. In fact, as commented in the introduction, MARLS could utilize HS binding to gain entry into cells through caveolae.

**Table 1:** Inducible depletion of PI(4,5)P_2_ from plasma membrane inhibits internalization of FMDV and VSV.

		% Cells with internalized virions	% Cells without internalizedvirions	P-value
CS8-c1	Control	89	11	0.0001*
	Rapamycin	12	88	
MARLS	Control	83	17	0.2202
	Rapamycin	76	24	
VSV	Control	88	12	0.0001*
	Rapamycin	15	85	

Number of cotransfected cells scored: 100.

* Statistically significant difference.

**Figure 5:**
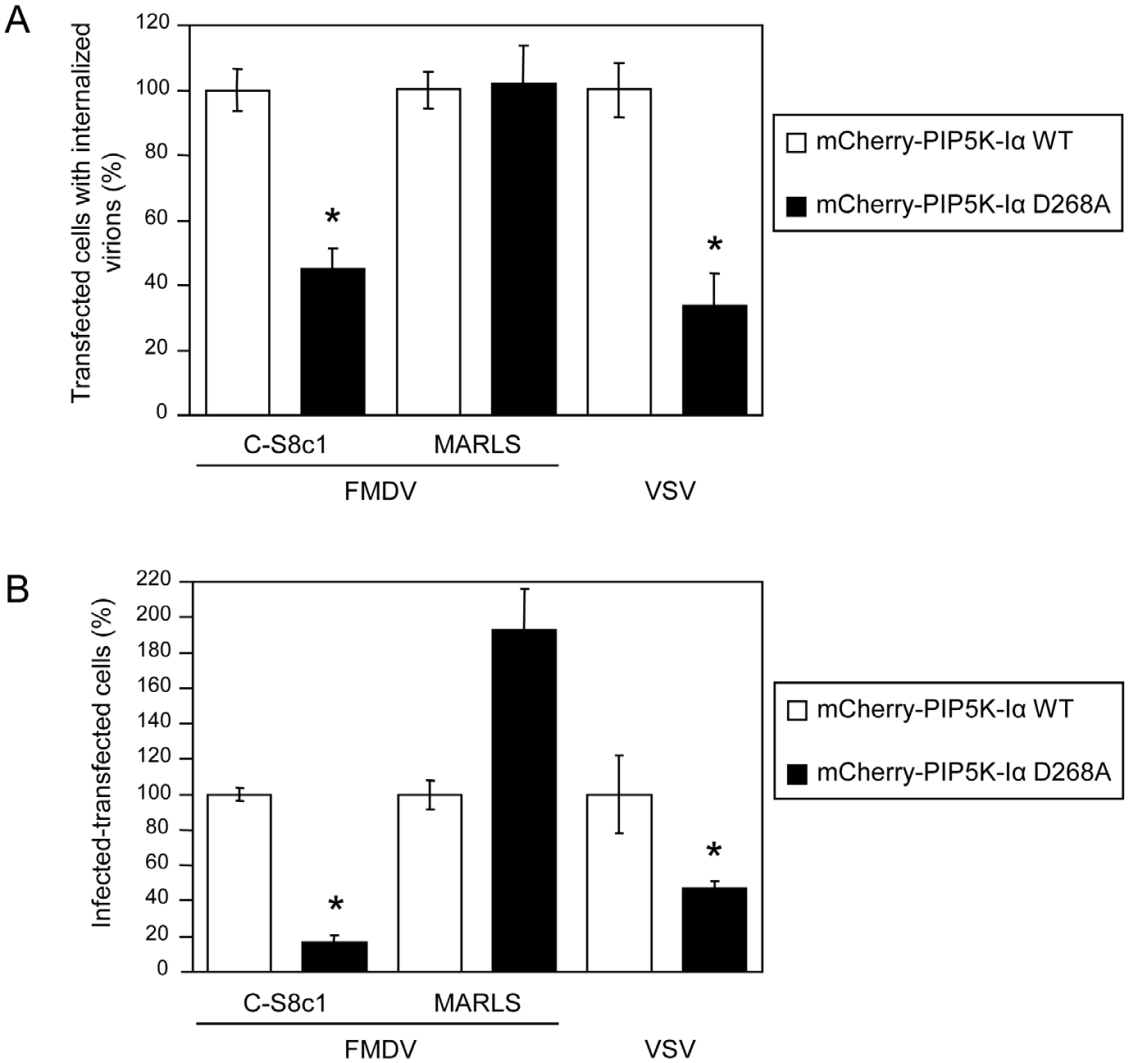
PIP5K-Iα is involved on entry and infection of FMDV C-S8c1 and VSV. (A) BHK-21 cells transfected with mCherry fused to WT or a KD version of PIP5K-Iα (mCherry-PIP5K-Iα WT and mCherry-PIP5K-Iα D268A, respectively) and 24 h later were incubated with the different FMDV variants (C-S8c1 and MARLS) or VSV (MOI of 70 PFU/cell) for 25 min and processed for immunofluorescence. The graph represents the percentage of cells that showed internalized virus determined as described in Materials and Methods. At least 100 transfected cells per coverslip were scored for each assay (3 coverslip). (B) BHK-21 cells were electroporated with a plasmid encoding mCherry-PIP5K-Iα WT as control, or mCherry-PIP5K-Iα D268A. At 24 h post-electroporation, monolayers were infected with the corresponding virus (MOI of 1 PFU/cell) and cells were fixed and processed for immunofluorescence at 7 h post-infection. Bars represent the mean percentage of transfected and infected cells ± SD, normalized to the level of infection of cells expressing the mCherry-PIP5K-Iα WT. Statistically significant differences between cells transfected with mCherry-PIP5K-Iα WT or D268A are indicated by an asterisk (ANOVA P≤0.05).

### PIP5K-Iα is Required for Efficient FMDV C-S8c1 and VSV Entry and Infection

Three distinct isoforms of PIP5K-I (α, β, and γ) are responsible for the synthesis of a major proportion of PI(4,5)P_2_ in the cell, through the hosphorylation of PI4P [13]. Among these enzymes, PI4P5K-Iα has been identified as the main isoform that regulates clathrin-dependent endocytosis [2], [55]., and expression of kinase dead (KD) or truncated mutants of PIP5K-Iα impairs clathrin mediated endocytosis [55]. In this regard, the role of this kinase on the internalization of FMDV (C-S8c1 and MARLS) and VSV was addressed by studying the effect of the expression of a KD mutant of PIP5K-Iα fused to mCherry protein – mCherry-PIP5KIα D268A [2] – on the entry and infection of these viruses. BHK-21 cells were transfected, incubated with the viruses, processed and analyzed by confocal microscopy as described [32], [48]. The percentages of cells expressing KD PIP5K-Iα that internalized VSV or C-S8c1 were significantly lower than those of control cells, being the reduction values of 66% and 55%, respectively (Fig. 5A). On the other hand, expression of KD PIP5K-Iα did not reduce the percentage of MARLS viral particles internalized by cells when compared with control samples (Fig. 5A). In addition to this, expression of KD PIP5K-Iα significantly reduced the percentage of transfected-infected cells for C-S8c1 (83% reduction) and VSV (53% reduction) (Fig. 5B). Conversely, in the case of MARLS an increase in the percentage of transfected-infected cells was observed upon expression of KD PIP5K-Iα. These results indicate the functional requirement of the PIP5K-Iα for the entry and infection of C-S8c1 and VSV, but not for MARLS. Even when further work is required to understand the infection increase caused by KD PIP5K-Iα on MARLS infection, these differences support the notion that MARLS is internalized using of an alternative dynamin-dependent non-clathrin endocytic pathway less affected by modulation of plasma membrane PI(4,5)P_2_ and independent on PI4P5K-Iα function.

Considering these results, modulation of PI(4,5)P_2_ metabolism, and of PIP5K function (especially PIP5K-Iα) could potentially constitute a new antiviral approach to fight viral diseases, a concept which has been already proposed for HIV-1 [21]. However, acute depletion of this lipid from plasma membrane has different effects on cellular functions [45], thus constituting a major concern for its potential application *in vivo*. Nevertheless, the experience with a variety of PI kinases that regulate synthesis of different PI species has revealed that these enzymes, and the lipids they synthesize, constitute good druggable targets to treat diverse diseases [56], including viral infections [57], [58], [59], [60], cancer [61], [62] or diabetes [63]. In particular, specific isoforms of PI 4-kinases can be chemically inhibited resulting in a blockage of viral replication without having a significant impact on cell viability [64]. As PI(4,5)P_2_ requirement is expected to be shared by a wide variety of viruses that are internalized using clathrin-mediated endocytosis, successful intervention on this pathway could lead to the development of broad spectrum antivirals. This concept, already proposed for PI 4-kinases, could offer therapeutic advantages since inhibition of host components instead of viral components could circumvent the problem of rapid selection of drug-resistant viruses – for a discussion see [64] –. Indeed, modulation of the metabolism of specific lipids is currently raising as a feasible antiviral approach [65], [66], [67]. Overall, the results presented in this study highlight the involvement of PI(4,5)P_2,_ on viral entry of either enveloped and non-enveloped viruses. Further work has to be performed to evaluate the feasibility of the depletion of PI(4,5)P_2_ from plasma membrane as a novel antiviral strategy.

## Materials and Methods

### Cells and Viruses

BHK-21 cells (ATCC) were grown in Dulbecco’s modified Eagle’s medium (DMEM) supplemented with 5% fetal calf serum (FCS), L-glutamine (2 mM), penicillin (100 U/ml), and streptomycin (100 µg/ml). FMDV isolate C-S8c1 is a derivative of a type C field virus isolated in Santa Pau (Spain, 1970) by triple plaque purification [68]. MARLS virus is a monoclonal antibody (MAb)-resistant mutant isolated with MAb SD6, which recognizes the G-H loop of capsid protein VP1 [69], from C-S8c1 virus after 213 passages on BHK-21 cells [36]. Mutations in MARLS virus compared to parental C-S8c1 have described previously [29]. VSV Indiana [70] was also used. The sequence of the capsid proteins of C-S8c1 and MARLS stocks used in this work was confirmed by RT-PCR amplification of viral RNA and sequencing of the amplicons obtained as described [71].

### Antibodies and Reagents

FMDV VP1 and VSV glycoprotein (G) protein were detected using MAb 5C4 [72] and I1 [73], respectively. Goat anti-mouse IgG labelled with Alexa Fluor (AF) 555 or 647 were from Molecular Probes. Transferrin (TF) conjugated to AF 488 was from Invitrogen. Ionomycin (Sigma) and rapamicyn (Calbiochem) were prepared in DMSO as 1.4 mM and 1.1 mM stock solutions, respectively. 1-butanol and 2-butanol were from Merck.

### Drug Treatments

BHK-21 cells grown on coverslips were washed twice with DMEM and incubated with ionomycin (5 µM) for 30 min, or with 1.5% 1-butanol or 2-butanol for 5 min. Control cells were incubated in the same conditions in DMEM containing the solvent concentration used for each drug. The drug was maintained during the virus internalization time. Cell viability upon drug treatments was determined by propidium iodide staining and flow cytometry [74], [75] using a FACScalibur flow cytometer (Becton Dickinson).

### Plasmids and Transfections

The following plasmids were used in this study: eGFP-Dyn WT eGFP-Dyn K44A [47], PH-PLC-eGFP [44], PM-FRB-CFP, mRFP-FKBP-dom5ptase [45], mCherry-PIP5K-Iα WT and KD mCherry-PIP5K-Iα D268A [2] (all the PIP5K isoform designations in the text refer to the human nomenclature for these genes). BHK-21 cells were transfected using Lipofectamine Plus (Invitrogen) as described by the manufacturer or electroparated with the corresponding plasmid using Gene Pulser XCell™ (Bio Rad).

### Immunofluorescence

Immunofluorescence was performed as described previously [76]. For confocal microscopy; a LSM510 META Inverted (Zeiss) confocal laser scanning microscope coupled to an Axiovert200 (Zeiss) inverted microscope (objective Plan-Apochromat 63x/AN 1.4) was used. Images were acquired using Zeiss LSM510 4.2 Sp2 software. The percentage of cells which internalized viral particles was determined by observation of Z-stacks (scan zoom 1×, step size 0.4 µm) (n ≥100) of cells using confocal microscopy [77]. For conventional fluorescence microscopy an Axioskop (Zeiss) fluorescence microscope coupled to a Coolsnap FX monochrome camera Roper Scientific was used and were acquired using RS Image software (Roper Scientific). To determine the number of infected cells, more than 150 cells expressing GFP were analyzed and the experiment was carried out three independent times. The images were processed using Adobe Photoshop 7.0 (Adobe System Inc.).

### Data Analysis

Analysis of variance (ANOVA) using *F* Fischer-Snedecor distribution was performed with statistical package SPSS v.17.0 (SPSS Inc) for Windows. Data are presented as means ± standard deviations (SD). Chi-square test was performed with statistical package Graph Pad Prism. Statistically significant differences are denoted in the figures by one asterisk for a *P* value of <0.05.

## Supporting Information

Figure S1 **Analysis of cellular viability upon drug treatments.** Cellular viability upon treatment with ionomycin (A) or 1 and 2-butanol (B) was determined by propidium iodide staining and flow cytometry. Control cells were treated in parallel with drug vehicles.
(TIF)

Figure S2 **Inducible depletion of PI(4,5)P_2_ from plasma membrane inhibits internalization of FMDV and VSV.** BHK-21 cells were cotransfected with PM-FRB-CFP – indicated as CFP (blue) – and mRFP-FKBP-dom5ptase – indicated as RFP (red) – plasmids [45]. At 24 h post-transfection, cells were treated (right panels) or not (left panels) with 10 nM rapamycin (10 min) to induce the depletion of PI(4,5)P_2_ from plasma membrane. Then, cells were incubated with the different viruses (green) (MOI of 70 PFU/cell, 25 min) in the presence of rapamycin and cells were processed for immunofluorescence. The percentage of cells that showed internalized virions, determined as described in Materials and Methods, is indicated. White dashed lines indicate the cell periphery of cotransfected cells; white solid lines indicate the cell periphery of untransfected cells. Insets show the lasser lines corresponding to the fluorochromes expressed by each of the transfecting plasmids, as well as a DIC image depicting the shape of the cells present in each field. Bar: 10 µm.
(TIF)
