# Supplementary figures and images for: Plasma Membrane Phosphatidylinositol 4,5 Bisphosphate Is Required for Internalization of Foot-and-Mouth Disease Virus and Vesicular Stomatitis Virus
Source: PLoS One. 2012 Sep 28;7(9):e45172. doi: 10.1371/journal.pone.0045172 (PMC3460999; doi:10.1371/journal.pone.0045172)

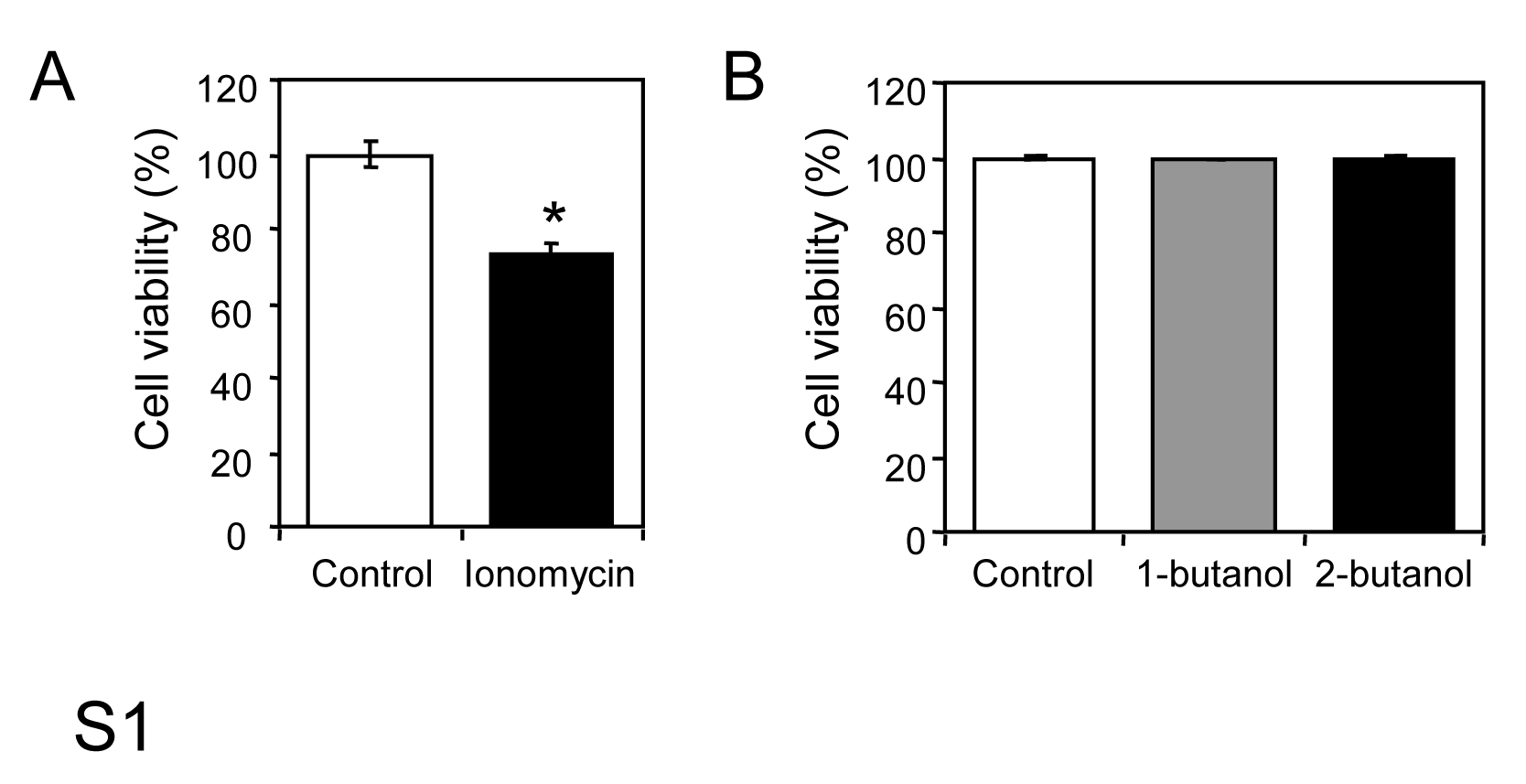

Supplement: Figure S1 — Analysis of cellular viability upon drug treatments. Cellular viability upon treatment with ionomycin (A) or 1 and 2-butanol (B) was determined by propidium iodide staining and flow cytometry. Control cells were treated in parallel with drug vehicles. (TIF) [file pone.0045172.s001.tif]

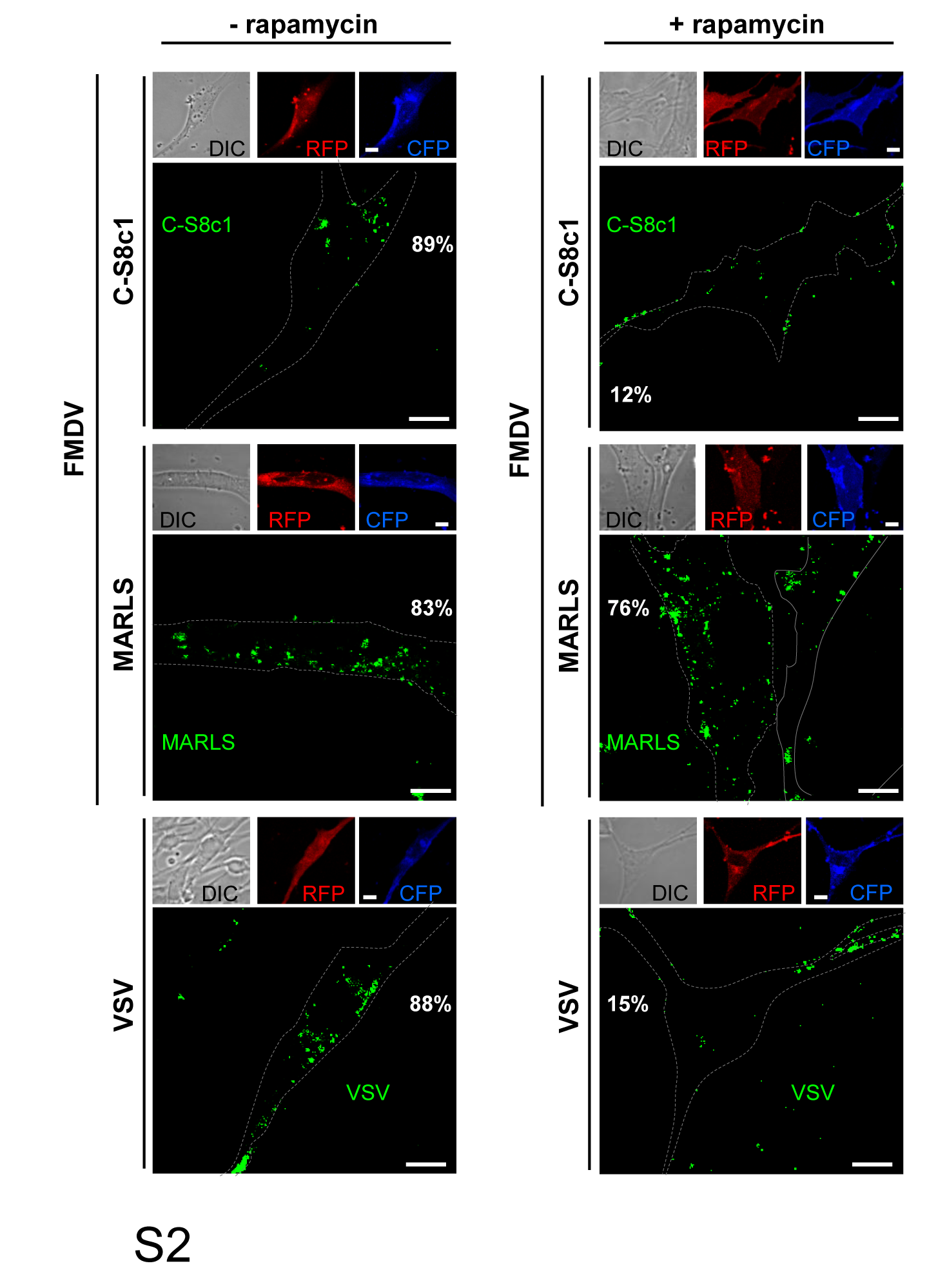

Supplement: Figure S2 — Inducible depletion of PI(4,5)P2 from plasma membrane inhibits internalization of FMDV and VSV. BHK-21 cells were cotransfected with PM-FRB-CFP – indicated as CFP (blue) – and mRFP-FKBP-dom5ptase – indicated as RFP (red) – plasmids [45]. At 24 h post-transfection, cells were treated (right panels) or not (left panels) with 10 nM rapamycin (10 min) to induce the depletion of PI(4,5)P2 from plasma membrane. Then, cells were incubated with the different viruses (green) (MOI of 70 PFU/cell, 25 min) in the presence of rapamycin and cells were processed for immunofluorescence. The percentage of cells that showed internalized virions, determined as described in Materials and Methods, is indicated. White dashed lines indicate the cell periphery of cotransfected cells; white solid lines indicate the cell periphery of untransfected cells. Insets show the lasser lines corresponding to the fluorochromes expressed by each of the transfecting plasmids, as well as a DIC image depicting the shape of the cells present in each field. Bar: 10 µm. (TIF) [file pone.0045172.s002.tif]
